# An Angiotensin II (Ang II) Type 1 Receptor Blocker, Telmisartan, Improves Insulin Resistance in KK-A^y^ Diabetic Mice

**Published:** 2006-12

**Authors:** Toshiyuki Takasu, Hirotoshi Kakuta, Masao Sasamata, Sho-ichi Yamagishi

**Affiliations:** 1 *Applied Pharmacology II Pharmacology Research Laboratory, Drug Discovery Research, Astellas Pharma Inc., Japan;*; 2 *Department of Internal Medicine, Kurume University School of Medicine, Kurume, Japan*

**Keywords:** telmisartan, insulin resistance, hypertension, diabetes, PPAR-γ

## Abstract

Metabolic syndrome is strongly associated with insulin resistance and consists of a constellation of factors such as hypertension and hyperlipidemia that raise the risk for cardiovascular diseases and diabetes mellitus. The renin-angiotensin system (RAS) plays a pivotal role in the pathogenesis of diabetes and cardiovascular disease (CVD) in hypertensive patients. Further, recently, the interruption of the RAS has been shown to prevent the onset of diabetes in hypertensive patients. However, whether telmisartan, an angiotensin II type 1 receptor blocker (ARB) with selective peroxisome proliferator-activated receptor-γ (PPAR-γ) agonistic property could improve insulin sensitivity is not fully understood. In this study, we studied the effects of telmisartan on insulin sensitivity in KK-A^y^ mice, an obese type 2 diabetic animal. Although there was no significant difference in body weight, food consumption, and glucose levels between the two groups, plasma insulin, triglycerides and non-esterified fatty acid levels were significantly decreased in telmisartan-treated KK-A^y^ mice, compared with control KK-A^y^ mice. The present findings suggest that telmisartan could exert a beneficial effect on insulin sensitivity in diabetic animals. Inhibition of the RAS by telmisartan, a selective agonist of PPAR-γ, may become a promising strategy for the treatment of hypertensive patients with metabolic syndrome and/or insulin resistance.

## INTRODUCTION

Metabolic syndrome is strongly associated with insulin resistance and consists of a constellation of factors such as hypertension and hyperlipidemia that raise the risk for cardiovascular diseases and diabetes mellitus (1). Hypertension occurs approximately twice as frequently in patients with diabetes compared with in non-diabetic controls (2-5). Conversely, recent data suggest that hypertensive patients are more likely to develop diabetes than normotensive persons (2-5). The association of diabetes with hypertension increases its risk of cardiovascular morbidity and mortality. Indeed, up to 75% of cardiovascular disease (CVD) in diabetic patients can be attributed to hypertension (2-5). Therefore, the primary goals for the treatment of metabolic syndrome and/or insulin resistant are the prevention of type 2 diabetes and cardiovascular events.

There is a growing body of evidence that the renin-angiotensin system (RAS) plays a pivotal role in the pathogenesis of diabetes and CVD in patients with hypertension (6-9). Indeed, interruption of the RAS with angiotensin-coverting enzyme inhibitors (ACEIs) or angiotensin II type 1 receptor blockers (ARBs) has been recently shown to prevent the onset of diabetes in hypertensive patients and to reduce cardiovascular and renal disease progression in diabetic patients with hypertension (6-9). These observations suggest that inhibition of the RAS could be a promising therapeutic strategy for the treatment of hypertensive patients with the metabolic syndrome and/or insulin resistance. However, whether telmisartan, an ARB with selective peroxisome proliferator-activated receptor-γ (PPAR-γ) agonistic property, could improve insulin sensitivity is not fully understood. In this study, we studied the effects of telmisartan on insulin sensitivity in KK-A^y^ mice, an obese type 2 diabetic animal model (10, 11).

## MATERIALS AND METHODS

### Drugs

Telmisartan was provided by Nippon Boehringer Ingelheim (Kawanishi, Hyogo, Japan).

### Animals

Seven week-old male KK-A^y^ mice were purchased from Clea Japan (Tokyo, Japan). They were housed in individual animal cages under a controlled temperature of 23°C ± 2°C and with a light period between 7:30 and 20:30 during the study period. They were allowed free access to water and standard CMF diet, which contains 29.0% proteins with vitamin and mineral mixture (372 kcal/100g) (Oriental Yeast, Co., Ltd., Tokyo, Japan). All animal experiments were performed in accordance with Guiding Principles for the care and use of laboratory animals approved by the Japanese Pharmacological Society. The ethical committee of Astellas Pharma Inc. approved this study.

After measurement of body weight and systolic blood pressure (BP), animals were allocated into two groups: (A) control (N=10) and (B) telmisartan 10 mg/kg (N=10). Telmisartan (B) or 0.5% methylcellulose solution (A) was orally administered for 2 weeks in KK-A^y^ mice.

### Clinical parameters

Animals were placed in metabolic cages. Blood samples were taken from jugular veins of each mouse at one-week interval for the measurement of blood glucose, plasma insulin, triglycerides (TG), and non-esterified fatty acid (NEFA). Blood chemistries were measured enzymatically with commercially available kits (Wako Chemicals, Osaka, Japan for glucose, TG and NEFA measurements; Amersham Biosciences Co., Ltd., Tokyo, Japan for insulin measurement).

### Statistical analysis

All results were analyzed using Statistical Analysis System (SAS Institute Inc, NC, USA). Data were shown in mean ± SD. Student’s t-test was used to analyze the effects of telmisartan on body weight, food consumption, and blood chemistries.

## RESULTS

**Figure 1:**
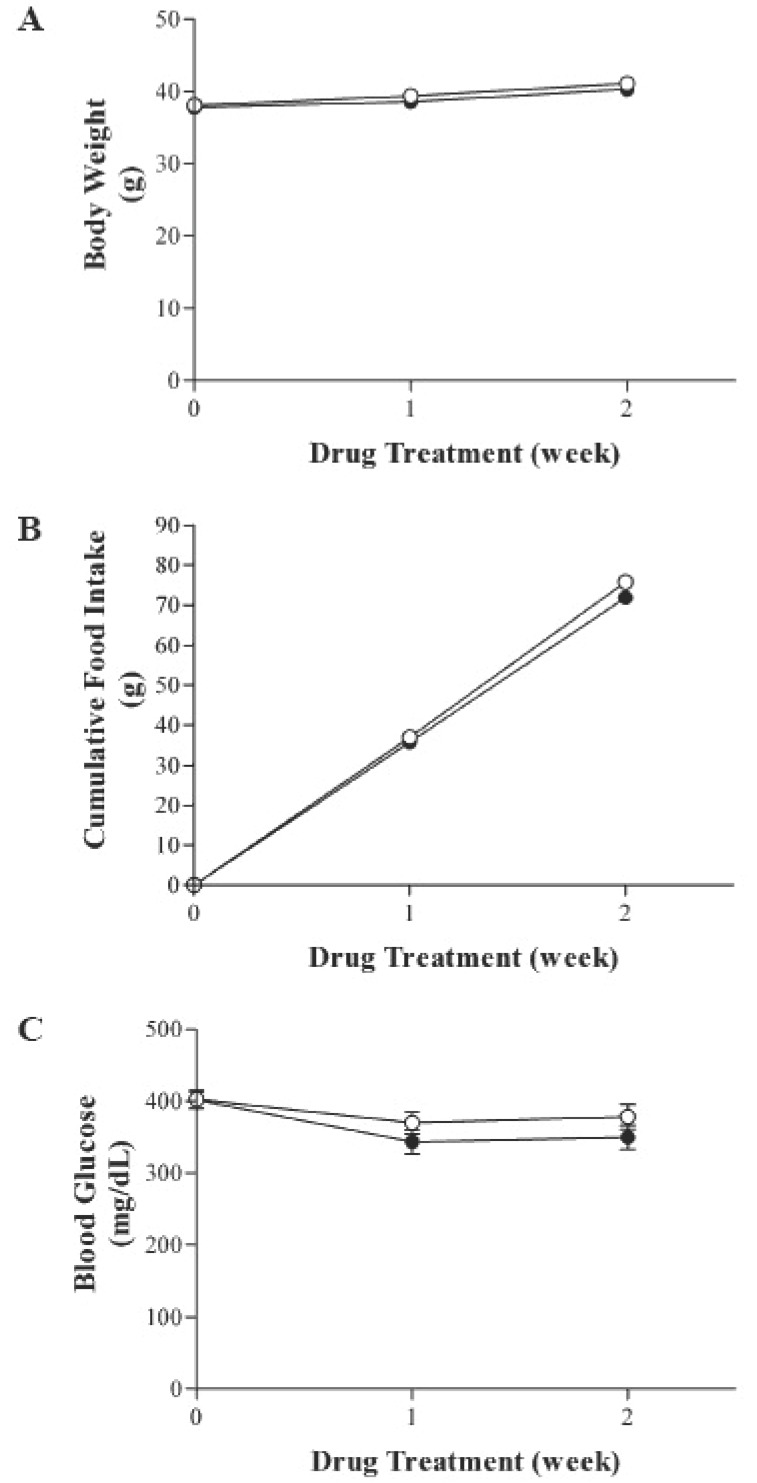
Effect of telmisartan treatment on body weight, food consumption and glucose levels in KK-A^y^ mice. Values represent means ± SD; ○, control (N=10); telmisartan (10 mg/kg) (N=10).

**Figure 2:**
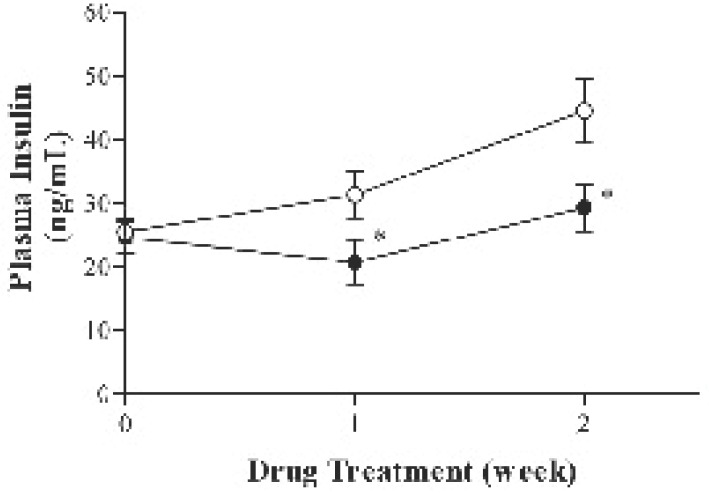
Effect of telmisartan treatment on plasma insulin levels in KK-A^y^ mice. Values represent means ± SD; ○, control (N=10); ●, telmisartan (10 mg/kg) (N=10). *P<0.05, vs. control group.

**Figure 3:**
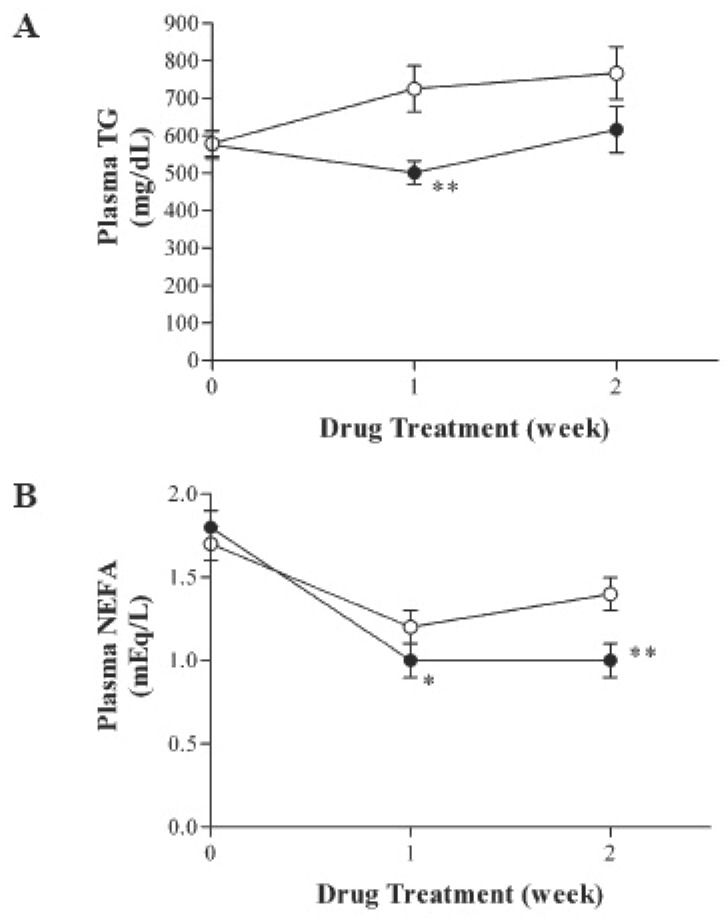
Effect of telmisartan treatment on plasma TG and NEFA levels in KK-A^y^ mice. Values represent means ± SD; ○, control (N=10); ●, telmisartan (10 mg/kg) (N=10). *P<0.05, **P<0.01, vs. control group.

During the entire study period all animals gained weight normally. As shown in Fig. 1, there was no significant difference in body weight, food consumption and blood glucose levels between the two groups. However, plasma insulin levels were significantly decreased in telmisartan-treated KK-A^y^ mice (Fig. 2). Further, oral administration of telmisartan for 2 weeks significantly reduced the plasma levels of TG and NEFA in KK-A^y^ mice (Fig. 3).

## DISCUSSION

There is accumulating evidence that the RAS plays a pivotal role in the pathogenesis of diabetes (6-9). Indeed, large clinical trials have demonstrated substantial benefit of the blockade of this system for preventing the onset of diabetes in hypertensive patients (6-9). However, since Ang II-type 1 receptor interaction down-regulates peripheral blood flow in skeletal muscles, it remains still unclear whether blood pressure (BP) lowering-independent effects of this class of agents, that is, pleiotropic effects, could partly contribute to the improvement of insulin sensitivity in hypertensive patients. In this study, although plasma glucose levels remained unchanged during the experiments, short-term treatment of telmisartan (2 weeks) decreased plasma insulin, TG, and NEFA levels in KK-A^y^ mice, an obese type 2 diabetic animal model. Since we have very recently found that oral administration of 10 mg/kg telmisartan (the same dosage used here) for 2 weeks does not affect BP levels in spontaneously hypertensive rats stroke-prone infused with Ang II (unpublished data), our present observations suggest that telmisartan may improve insulin sensitivity in these animals partly in a BP-independent manner.

Recently, telmisartan was found to act as a partial and selective agonist of PPAR-γ, thus reducing glucose, insulin, and TG levels in rats fed a high-fat, high-carbohydrate diet (12, 13). PPAR-γ influences the gene expression involved in carbohydrate and lipid metabolism, and pioglitazone and rosiglitazone, ligands for PPAR-γ, improve insulin resistance in diabetic patients. These observations suggest that the insulin-sensitizing property of telmisartan observed here may be ascribed, at least in part, to its unique PPAR-γ-modulating activity. There is several papers to show that activators of PPAR-γ could also exert anti-inflammatory, anti-oxidative and anti-proliferative effects on vascular wall cells, thus decreasing the risks for atherosclerosis (14, 15). Inhibition of the RAS by telmisartan, a selective agonist of PPAR-γ, may become a promising strategy for the treatment of hypertensive patients with the metabolic syndrome and/or insulin resistance.
